# Physical activity and *beverage* consumption in preschoolers: focus groups with parents and teachers

**DOI:** 10.1186/1471-2458-13-278

**Published:** 2013-03-27

**Authors:** Marieke De Craemer, Ellen De Decker, Ilse De Bourdeaudhuij, Benedicte Deforche, Carine Vereecken, Kristin Duvinage, Evangelia Grammatikaki, Violeta Iotova, Juan Miguel Fernández-Alvira, Kamila Zych, Yannis Manios, Greet Cardon

**Affiliations:** 1 Department of Movement and Sport Sciences, Faculty of Medicine and Health Sciences, Ghent University, Watersportlaan 2, Ghent 9000, Belgium; 2 Department of Human Biometry and Biomechanics, Vrije Universiteit Brussel, Pleinlaan 2, Brussels 1050, Belgium; 3 Department of Public Health, Ghent University, De Pintelaan 185, Block B, Ghent 9000, Belgium; 4 Research Foundation – Flanders, Egmontstraat 5, Brussels 1000, Belgium; 5 University of Munich Medical Centre, Dr. von Hauner Children’s Hospital, Lindwurmstraße 4, Munich 80337, Germany; 6 Department of Nutrition and Dietetics, Harokopio University, E. Venizelou 70, Athens 17671, Greece; 7 Clinic of Paediatric Endocrinology, Medical University Varna, UMHAT “St. Marina”, 1 “Hr. Smrinenski” Blvd, Varna 9010, Bulgaria; 8 GENUD (Growth, Exercise, Nutrition and Development) Research Group, University of Zaragoza, C/Corona de Aragón, 42, 2ª planta, Zaragoza 50009, Spain; 9 Children’s Memorial Health Institute, Al. Dzieci Polskich 20, Warsaw 04 730, Poland

**Keywords:** Physical activity, *Beverage consumption*, Preschool children, Qualitative study, European study, ToyBox-study

## Abstract

**Background:**

Qualitative research is a method in which new ideas and strategies can be discovered. This qualitative study aimed to investigate parents’ and teachers’ opinions on physical activity and *beverage consumption* of preschool children. Through separate, independent focus groups, they expressed their perceptions on children’s current physical activity and *beverage consumption* levels, factors that influence and enhance these behaviours, and anticipated barriers to *making* changes.

**Methods:**

Multi-cultural and multi-geographical focus groups were carried out in six European countries (Belgium, Bulgaria, Germany, Greece, Poland and Spain). In total, twenty-four focus groups with 122 parents and eighteen focus groups with 87 teachers were conducted between October 2010 and January 2011. Based on a semi-structured interview guide, questions on preschoolers’ physical activity (opinions on preschoolers’ physical activity, how to increase physical activity, facilitators and barriers of physical activity) and *beverage consumption* (rules and policies, *factors influencing promotion of* healthy drinking, recommendations for future intervention development) were asked. The information was analyzed using qualitative data analysis software (NVivo8).

**Results:**

The focus group results indicated misperceptions of caregivers on preschoolers’ physical activity and *beverage consumption* levels. Caregivers perceived preschoolers as sufficiently active; they argue that children need to learn to sit still in preparation for primary school. At most preschools, children can drink only water. In some preschools sugar-sweetened beverages like chocolate milk or fruit juices, are also allowed. It was mentioned that sugar-sweetened beverages can be healthy due to mineral and vitamin content, although according to parents their daily intake is limited. These opinions resulted in low perceived needs to change behaviours.

**Conclusions:**

Although previous research shows need of change in obesity-related behaviours, the participants in the current study didn’t perceive such. The awareness of parents and teachers needs to be raised concerning their shared responsibility about healthy behaviours in preschoolers. Providing preschool teachers with ready-to-use classroom material will encourage them to *change* physical activity and beverage consumption, and to implement related activities in the classroom. Involvement in activities that their children perform at preschool will motivate parents to extend these behaviours to the home environment.

## Background

In developed countries, the prevalence of overweight and obesity among children between the age of zero and five years increased from 7.9% in 1990 to 11.7% in 2010 with an expected prevalence of 14.1% in 2020 [1]. This increase in obesity prevalence is driven by changes in lifestyle *(behaviour)* rather than changes in biologic or genetic factors [2]. The cumulative effect of eating behaviour, sedentary behaviour and physical activity (PA), referred to as energy balance-related behaviours (EBRBs), determines whether a positive energy balance or weight gain is experienced [3]. Consequently, to prevent this unnecessary weight gain, interventions should target EBRBs [4]. In the scope of obesity prevention, focusing on one single EBRB as a universal causal factor in obesity, is not ideal [3] as focusing on these EBRBs simultaneously could be more effective in the prevention of overweight in preschoolers [5].

Some reviews on *physical activity and diet* behaviours, such as in preschoolers, have already been published. Recent reviews and studies concluded that activity patterns of preschoolers are characterized *by low levels of total PA*[6-9]. *For example, in a study by Hinkley et al., virtually no preschool children (<1% of preschoolers) met the recent Australian PA guidelines of 180 minutes of PA per day*[9]*.* Several studies in different countries showed low intakes of non-sugared beverages, fruit, vegetables, water and milk and high intakes of unhealthy snacks, sugared drinks, juices, total and saturated fat and added sugar [10-15]. A *growing* number of studies on behaviours and determinants of PA and *beverage consumption* in preschool children have already been published [10-13,16-18]. On the other hand, studies on the perspectives of the caregivers (i.e. parents and teachers) on how to change these behaviours (i.e. more PA and water consumption) in the scope of overweight and obesity prevention are currently lacking.

Qualitative research – and in particular the execution of focus groups – is a research method in which new information and ideas can be discovered, which would not be possible with questionnaires. Conducting focus groups is a good step in collecting ideas and strategies for future interventions. For PA and *beverage consumption*, focus groups with parents, teachers or caregivers have already been conducted to obtain extra information on *these specific behaviours*, performed by the preschoolers at home or at preschool. These focus groups investigated barriers (e.g. trouble finding enough time to help the children, space, equipment, safety, inclement weather), and facilitators (e.g. parent role modelling, parent support, a child’s preference for being active, organised activities) of children’s PA and parental feeding styles, preschool nutrition policies and practices, and barriers and facilitators of healthy eating for preschoolers’ nutrition [19-32]. However, no focus group discussions have ever assessed recommendations of these caregivers (parents and teachers) for future intervention development, and what they perceive as feasible strategies.

Involving the parents is important, because the home environment can be considered as the most important place where children develop these behaviours [22,33]. Nonetheless, preschool-aged children also spend a considerable amount of time at preschool educational programs *and many children attend preschool*: about 99% of *all preschool-aged children attend preschool* in Belgium, about 98% in Spain, about 95% in Germany, about 70% in Bulgaria, and about 45% in Greece and Poland [34,35]. Consequently, such setting could as well play an important role in the promotion of healthy EBRBs in preschool children.

To our best *of our* knowledge, only one other study previously investigated parental perceptions on suggestions for PA programming in preschoolers [36]. No other studies have been carried out, focusing on parents’ and teachers’ recommendations for future intervention development and feasible strategies to increase PA and *change beverage consumption* in preschoolers. *Therefore, the aim of this study is to qualitatively assess parents’ and teachers’ perceptions of children’s PA and beverage consumption behaviours, factors that influence these behaviours, strategies to enhance these behaviours, and anticipated barriers to change.* More insight *into parent and teacher* perceptions can help to develop a more effective intervention in preschoolers that aims at increasing preschoolers’ PA and *modifying beverage consumption*. This method follows the community-based participatory research methodology, in which interventions are developed by a bottom-up approach [37].

Furthermore, this paper investigates these opinions on a European level, by executing focus groups in six different European countries. The execution of these multi-country focus groups on a cross-European level adds multi-cultural and multi-geographic value to the results found in the focus group discussions. Based on the outcomes of this study, a European preschool-based intervention – named ToyBox – has been developed.

## Methods

### Study background

This qualitative study was carried out as part of the European ToyBox-study, and was implemented in the preschool setting. The ToyBox-study is a multifactorial evidence-based approach to prevent overweight and obesity in preschool children between the age of four and six years old [38]. The project targets: (1) increasing PA, (2) decreasing sedentary behaviour, (3) increasing the water intake and (4) decreasing the intake of sugar-sweetened beverages. Within the scope of this study, focus groups with parents and teachers of four- to six-year-old preschoolers were conducted. In this paper, *findings* on PA and *beverage consumption* will be reported. *For this paper, we will use the term “preschool” to refer to the range of preschool educational programs (e.g. preschool, childcare, or kindergarten) which are available, in one form or another, in all six European countries*[39]. This study was included in the approval of the ToyBox-study by Ethical Committees in all six European countries, in line with national regulations (i.e. the Ethical Committee of Ghent University Hospital (Belgium), CEICA (Comité Ético de Investigación Clínica de Aragón (Spain), Ethikkommission der Ludwig- Maximilians-Universität München (Germany), Komisja Bioetyczna (Poland), Committee for the Ethics of the Scientific Studies (KENI) at the Medical University of Varna (Bulgaria), and the Ethical Committee of Harokopio University of Athens (Greece)).

### Participants

Between October 2010 and January 2011, focus groups were executed in six different European countries: Belgium, Bulgaria, Germany, Greece, Poland and Spain. The focus groups were conducted in municipalities with the highest prevalence of overweight or obesity of either child or parent, because the ToyBox intervention will take place in comparable municipalities. When this was not feasible, the prevalence of overweight had to be higher than the national level. In each country, four focus groups were undertaken with parents and three with teachers. In order to receive feedback from parents *across the range of* socioeconomic status (SES) - who will both be targeted in the ToyBox intervention - two focus groups were performed in parents of low SES (secondary school or less) and two focus groups in parents of medium or high SES (parents with higher education, college or university) in each country. An overview of the number of participants by low SES and medium-high SES can be found in Table 1. The recruitment of parents of children attending preschool and teachers mostly occurred through preschools and through the researchers’ networks.

**Table 1:** Overview of the number of participants by low SES parents and medium-high SES parents in six European countries (Belgium, Bulgaria, Germany, Greece, Poland, and Spain)

**Country**	**Number of low SES parents**	**Number of medium-high SES parents**
**Belgium**	6	10
**Bulgaria**	6	16
**Germany**	7	11
**Greece**	8	13
**Poland**	8	10
**Spain**	11	16
**Total**	46	76

### Procedure

#### Logistics

Focus groups took place at preschools or local meeting rooms that were easily accessible. The focus groups lasted from one hour up to two hours and took place in a sufficiently comfortable and neutral room. *At the onset, parents and teachers had to fill out a demographic questionnaire and a written informed consent – in which they gave permission to audio-tape the focus groups.* In all countries, refreshments and biscuits or fruit were provided for the participants. In three countries, incentives were given because of difficulties with the recruitment (fruit basket, cinema ticket).

#### Focus group procedures

All partners followed focus group training led by researchers from Belgium to ensure consistency among the different countries. To obtain standardization, a structured protocol - including a semi-structured interview guide - was developed reviewed, accepted and used in all participating countries.

The protocol consisted of guidelines for the sampling and recruitment of the participants, information about the location and setting, the taping of the discussions and guidelines for the moderator and co-moderator so they could optimally lead the sessions. The focus groups were led by a trained moderator and were assisted by a co-moderator. The moderator was familiar with the interview guide so that the topics for discussion could be introduced. Furthermore, the moderator helped the group to participate in a lively and natural conversation. The co-moderator handled the logistics by for example taking notes, monitoring the recording equipment, or arranging the tables/chairs in the room. After each focus group session, both moderator and co-moderator debriefed, *focusing on* the most important topics *raised*, different ideas, differences with previous focus groups, unexpected findings and main impressions about the session.

*The semi-structured interview guide for PA and beverage consumption was developed in accordance with established guidelines*[40-42]*, based on the themes of the ToyBox-project and on previous focus groups on PA or nutrition*[43,44]*. The interview guide was formulated to investigate the parents’ and teachers’ perspectives of influences on preschool children’s PA and beverage consumption. The questions provided were broad and open-ended. More detailed optional questions were asked when the discussion did not start up or continue spontaneously.*

### Data analysis

A verbatim written transcription of the focus groups in the local language was made in all countries, based on the information of the audiotapes. After each country *obtained* full transcripts of the focus group discussions, a qualitative content analysis of the transcriptions was independently conducted by local researchers in each country, based on the instructions in the standardized protocol (either with or without data analysis software) to standardize the analysis of the focus groups. The original information and main findings of the focus groups were identified and written down in English in a standardized template, including quotes and *excerpts from* the transcripts. In each country, this information was put into a report and was sent to the researchers *responsible*.

Two researchers analyzed and summarized the available information from the six reports (including quotes and exerts) from all six countries using the qualitative data analysis software NVivo8 (QSR International Pty Ltd., Doncaster Victoria, Australia, Version 8, 2008). A data framework to code the data was used and was based on the major topics of the interview guide. After analysing the data from all six countries, all findings from all countries were summarized into a covering report, and included quotes and *excerpts from* the transcripts. This report was reviewed and validated by all the focus group organizers [45,46].

## Results

In total, 24 focus groups with 122 parents and 18 focus groups with 87 teachers were conducted between October 2010 and January 2011. The total number of participants in all countries for parents ranged from 16 to 27 parents. For teachers, this number ranged from 11 to 20 participants. More detailed information is described in Table 2. An overview of the results can be found in Table 3 (PA) and Table 4 (*beverage consumption*).

**Table 2:** Descriptive information of the focus groups with parents and teachers in six European countries

	**Parents**			**Teachers**			
	**Range of participants (mean range)**	**Age range (mean age)**	**Total participants**	**Range of participants (mean range)**	**Age range (mean age)**	**Range of number of children in the classroom (mean number)**	**Total participants**
**BELGIUM**	2-6 (4.0)	30-45 (34.4)	16	2-8 (4.3)	22-55 (34.2)	14-30 (21.1)	13
**BULGARIA**	4-7 (5.5)	23-44 (35.1)	22	3-5 (4.0)	23-59 (46.5)	22-34 (28.4)	12
**GERMANY**	3-7 (4.5)	27-50 (36.8)	18	3-7 (4.7)	26-59 (42.9)	24-55 (29.8)	14
**GREECE**	4-7 (5.3)	35-44 (38.4)	21	5-6 (5.7)	29-52 (43.6)	17-25 (21.1)	17
**POLAND**	4-6 (4.5)	26-38 (32.0)	18	3-4 (3.7)	28-43 (35.5)	20-27 (22.2)	11
**SPAIN**	4-10 (6.8)	28-43 (36.3)	27	5-8 (6.7)	28-52 (41.4)	14-18 (16.0)	20
**TOTAL**			**122**				**87**

**Table 3:** Results from parents’ and teachers’ opinions on preschoolers’ physical activity

**Theme**	**Parents**	**Teachers**
**Opinion on preschoolers’ physical activity levels**	Preschoolers are sufficiently active.	Preschoolers are already very active. They also have to learn to sit still in preparation for primary school.
	It is not necessary to increase their physical activity level.	
**How to increase preschoolers’ physical activity levels**	Being a role model for the child.	Using the hallway, the dining-hall or other spaces to do movement activities.
	Playing together with the child.	Organizing a sports day or an “Olympic day”.
	Let the child participate in organized activities.	Let the children bring their bicycle, roller-skates or rollerblades to preschool.
	Regularly going outside with the children.	Morning gymnastics.
		Traditional-, balance- and team games.
**Facilitators to increase preschoolers’ physical activity**	Having friends and/or siblings; having cousins’ and/or neighbours’ children; preschool providing sports activities; having an environment which invites the children to be active; having acquaintances with children from the same age, size of the garden, space at home.	Available facilities, enough space, stimulating material, availability of the gym room
		Children’s reactions (smiling, having fun, being happy); parents’ approval; children’s joy of being allowed to experience things by themselves.
**Barriers to increase preschoolers’ physical activity**	Lack of time, not being in the mood to play together with the child, big distance to the sports club, work-load, cost price, means of transport.	Staff shortage
		Safety of the playground.
		Time schedule.
**Do you have recommendations for a future intervention targeting physical activity in preschoolers?**	Involving the parents in child-activities.	Ready-to-use material.
	Organization of parent–child activities.	Practical tips and information with new ideas and new activities.
		Teachers exchanging useful information to each other.
		Parental involvement.

**Table 4:** **Results from parents’ and teachers’ opinions on preschoolers’ *beverage consumption***

**Theme**	**Parents**	**Teachers**
**Beverages**	Children usually drink water, fruit juices (orange and apple juices), milk (plain, chocolate, flavoured/sweetened), soft drinks and (un)sweetened tea.	Most teachers think they have an important role in increasing preschoolers’ water intake (one of the main role models).
	A few parents (different countries) do not think it is necessary to decrease the intake of sugar-sweetened beverages (soft drinks and coloured milk).	Children usually drink water.
		In some preschools and countries, children drink milk (plain, chocolate, flavoured/sweetened), fruit juices and (unsweetened) tea.
**How to increase preschoolers’ water intake**	Being a role model for the child.	Remind them to drink water before the start of a long activity or after being physically active.
	Making it a habit to drink water at home.	When children are thirsty, they can always drink water in the classroom.
	Putting a water jug on the table, together with some glasses.	
	Providing a nice drinking cup or a bottle with a sports cap.	
**Barriers to increase preschoolers’ water intake**	Children tend to forget that they have to drink; children have to be reminded to drink more; parents cannot control the water access at preschool.	They have to go to the bathroom more frequently.
		Parents that think that teachers are meddling in their family situation.
		Not all parents want to introduce the preschool rules at home.
**How to decrease preschoolers’ intake of sugar-sweetened beverages**	Not buying those beverages; diluting soft drinks with water; using cacao powder instead of chocolate milk; using fresh fruits instead of packed fruit juices or fruit drinks; not drinking soft drinks themselves.	Soft drinks are not distributed at preschool.
		Only allow the intake of water.
**Do you have recommendations for a future intervention targeting *beverage consumption *in preschoolers?**	Having the children bring home information from preschool on *beverage consumption*.	Educate the parents.
		Ready-to-use material.
		Practical tips and information with new ideas and new activities.
		Teachers exchanging useful information to each other.
		Parental involvement.

### Physical activity

#### Parental and teacher perceptions of children’s current PA levels

The majority of parents think that their children are sufficiently active and do not need additional activity. Many parents reported that their preschool child is a member of a sports club. For example, preschoolers attend lessons in gymnastics, swimming, or dancing classes. Some parents raised concerns that children should not be involved in too many organized activities, so that they have sufficient time for resting as well.

“I rather think that I have to slow down my child at that age.”

"Mine is very active, so we wanted a big part of this energy to be channeled to a sport.”

“He should not get too much… he is only 4.”

The majority of the teachers mentioned that preschoolers are already very active. Although they think that sufficient PA is healthy and beneficial for the children, they also reported that it is important that preschool children also learn to sit still in preparation for primary school.

“It’s difficult for the children to sit still for a certain period of time.”

### Factors influencing children’s PA (facilitators and barriers)

Parents were able to identify multiple factors of the environment that either facilitated their child’s activity or created barriers to it, most of which were related to the physical environment. Almost all parents mentioned that the weather was the most important factor as it determines whether or not their child can play outside. Environmental factors that were perceived to encourage children’s PA included having a playground close to the house, living in a rural area (children have more space to play outside), having an allotment, garden or yard and living close to a forest to go for a walk. Environmental barriers to children’s PA having a TV left switched on, and having big streets that need to be crossed to reach the playground. In addition to these environmental factors, parental work-load was also perceived as a barrier.

“I do not have that much space in my house. My son cannot play upstairs in his room; there is only place for one bed and a closet. The only option I have, is letting my child play downstairs in a little corner.”

“We all work which means that we do not have enough free time for the children during the work week.”

Also teachers mentioned several aspects of the physical environment as influencing factors of children’s PA at preschool. Having enough available facilities and having nice weather during spring and summer facilitates the children to be more active at preschool. Other facilitators mentioned were having enough space at preschool, having stimulating material at their disposal to keep the children physically active, and having a gym room that is available to them. Reported barriers to children’s PA were staff shortage and safety of the playground.

“In order to offer moving opportunities, more staff is necessary. For instance, a climbing landscape requires more supervisory staff. The children are not allowed to do it by themselves.”

“We have a space… very big… thank God for the past 1.5 years we are in a new building, very nice and we have a lot of equipment…”

### Strategies to increase children’s PA at home and at preschool

While parents may not believe that their children need additional activity, they were confident that they would be successful in making their children more active if needed. They were able to come up with multiple strategies that could be employed. Parental strategies included being a role model for their child, going outside or playing outside together with their child, making a routine to regularly go outside, letting children participate in organized activities (e.g. swimming lessons, gymnastics for preschoolers) and making time to take the children to sports lessons on weekend days.

“I have decided to send my children to a youth movement or scouting to make them more physically active”

“When we are active, our children will also be active.”

One of the strategies mentioned by teachers that possibly could be used in the future to increase children’s PA at preschool was the use of other spaces, for example the playground, the hallway or the dining-hall. Furthermore, teachers reported they had already organised a sports day or an “Olympic day”. Other strategies used were: bringing their bicycle, roller-skates or rollerblades to preschool, organising morning gymnastics or playing traditional-, balance- and team games. Teachers mentioned that they are also role models for the children, when teachers are more active, the children will be more active as well.

“Sometimes the children are easier to motivate when I participate myself. When I run around and jump on one leg they have a lot more fun and rather take part than when I just stand there and play with my drum.”

#### Barriers in encouraging children to be more active

Parents also anticipated multiple barriers in encouraging their children to be more active. The most commonly reported barriers included not being in the mood to do activities together with their child, difficult access to PA areas, large distances to the sports club, high cost of activities, and means of transportation (having no car, having only one car, bicycles).

“The obstacle to play outside with the children is that I am too busy or lazy or something like that. However, playing outside by themselves, I think, is fine under appropriate circumstances.”

“It would be difficult for me. For example on a Sunday, that is my relaxation day. Most of the time, I am not in the mood to go for a walk or to go to a swimming pool. On that day, we just lie in our couch with our pyjamas on.”

For teachers, competition for resources and time were the major barriers reported. It is not always possible to use other spaces at the playground (e.g. playground, hallway) to make the children more active. For example, using the hallway is sometimes difficult because of the noise that is being produced and other classes could be disturbed.

### Motivational factors to make changes and overcome barriers

Parents must be motivated to encourage their children to be more active before they will invest in these strategies and tackle the anticipated barriers. Parents reported that a health professional or doctor’s advice to increase their child’s activity level would serve as a source of motivation. Additionally, a scientist offering information on the objectively measured PA level of their child could also help provide motivation and encouragement. Parents also reported that being involved in activities that the children perform at preschool could help motivate them to increase their child’s PA. Furthermore, when parent–child activities are organised – in which parents and children perform activities together – parents reported they would be more motivated to be involved in these activities.

Some teachers reported that they frequently hear children talk about watching television at home and the teachers reported believing that the children are inactive at home. For that reason, teachers want to promote PA at preschool.

“The last years, you hear that the children watch a lot of television. In the mornings, in the evenings, in the weekend,… I think it is important for the children to give them a chance to be physically active in the classroom.”

Other encouraging factors for promoting PA in preschoolers are the children’s reactions (smiling, having fun, being happy), parents’ approval and the children’s joy of being allowed to experience things by themselves. Also, when teachers received ready-to-use material, they would be more motivated to increase preschoolers’ PA levels. Furthermore, they mentioned that practical tips and new ideas and activities would be very helpful to them, as well as involving the parents into the classroom activities.

“Maybe some party games or books or stories would motivate us.”

“Encourage the parents to do their best, to participate.”

“It has to be feasible in the classroom and with the children. Not material for children older than six years, but really adjusted for the right age.”

### PA in six European countries (at home and at preschool)

Parental perceptions on preschoolers’ PA between European countries are not necessarily the same. German, Greek, Polish, Belgian and Spanish parents all agreed that preschoolers are already active enough. Only Bulgarian parents did not spontaneously mention anything about their children being physically active enough. More low SES parents compared to high SES parents did not perceive the need to increase the PA levels of their children.

According to Spanish teachers, preschoolers are already active enough and some teachers even mentioned that more PA would be too much because the children would be exhausted. On the other hand, in Bulgaria, teachers mentioned that preschool children are not active enough. Bulgarian teachers thought it was necessary to increase preschoolers’ PA levels. Furthermore, in Poland, Spain, Greece and Belgium, teachers mentioned that preschoolers are very energetic and cannot sit still in preschool. Only in Belgium and Spain, most children have two hours of physical education weekly. In Belgium, some children also have movement breaks in the classroom.

### Policies at European preschools

In the different European countries conducting these focus groups, differences across countries concerning policies on PA became clear. In Belgium, teachers reported they do not have a set policy on PA although they have a comprehensive policy on health and PA. In Bulgaria, teachers felt that the preschool policy focuses too much on teaching and other lessons, instead of on PA. In Greece, there are no set policies or rules, so teachers can set their own rules. In Poland, they do not have a policy on PA, while in Spain they have a policy but it varies across types of preschools (e.g. public (specific programs) versus private (more equipment)). In Germany, policies were not specifically mentioned, teachers talked more about the curriculum instead.

### Beverage consumption

#### Parental and teacher perceptions of children’s beverage consumption

The majority of the parents mentioned that their children usually drink all kinds of beverages like water, fruit juices (orange and apple juices), plain, chocolate and flavoured/sweetened milk, soft drinks and (un)sweetened tea. Some parents had established rules about the consumption of beverages. For example, preschoolers cannot drink soft drinks or only on special occasions or during the weekend but they are always allowed to drink water. Furthermore, preschool children have restricted access to fruit juices and chocolate milk (most of the time they can have it once a day) in some households. Finally, children cannot drink much before they eat or go to bed.

“My children only drink cartons of orange juice. I have nothing else.”

“No, I have no rules. My children only drink water and they can drink as much as they want!”

In Belgium, Germany and Greece, soft drinks are not allowed at preschool. Preschools are not allowed to provide or sell soft drinks and the children cannot bring them to preschool. In most preschools of these three countries, children can drink water, milk, chocolate milk, (organic) fruit juices and (unsweetened) tea. In Bulgaria, the children cannot bring beverages to preschool, they only have water available at the preschools. In Spain and Poland, only water is allowed.

In Bulgaria, Greece, Poland and Spain, the children can drink water, usually after permission from the teacher, throughout the day. Greek teachers reported that they remind children to drink water before starting a long activity or after being physically active. In Belgium, the children usually can drink together as a group at set times, for example before or after recess. Also, when some children are thirsty, they can always separately drink water. Teachers reported that they sometimes try to postpone the drinking to avoid that the children need to go to the toilet too often. Furthermore, in Poland, children get a glass of milk in the morning.

“We don’t have sugar sweetened beverages. We only offer water. The children drink water, milk, juice, diluted juices, and tea.”

#### Factors influencing children’s beverage consumption (at home and at preschool)

Parents mentioned that being a role model themselves influences their children’s beverage consumption. Next to playing a role themselves, almost all parents had the opinion that the preschool and the teachers also have a key role in the promotion of healthy drinks in young children. Nowadays, preschools and teachers pay attention to healthy eating and according to the parents, teachers are a role model for the children. Other parents mentioned that teachers do not have an influence on what their children drink. German low SES parents reported that the preschool and the teachers have a big influence on children concerning food.

“When children have chocolate milk or other sugared sweetened beverages at school every day, the preschool will have a stimulating and key role for the intake of sugar.”

Most teachers from all countries mentioned that they think they have an important role in increasing the water intake and decreasing the intake of SSBs in preschool children. Teachers think they are one of the main role models for preschool children’s consumption of beverages. Furthermore, they think it is important to educate the parents and to give them more information on this topic.

#### Strategies to improve children’s beverage consumption (intake of water and reduction of SSB)

Parents reported several strategies to increase the water intake of their children. First of all, the parents mentioned that they would try to be an example for the children by making it a habit to also drink water at home. Furthermore, they would put a water jug on the table, together with some glasses so that children can take and drink water whenever they want to. Finally, parents would also provide a nice drinking cup or a bottle with a sports cap, because this is also inviting for the children to drink water.

“The packaging is really important for children.”

“I think it would be a good idea to install such a water station or something like that. When the children get in contact with water right from the beginning they will get used to drinking water and unsweetened tea. Or maybe also fruit juices diluted with water or fruit-water mixes. I think it would be very important to install such a station.”

“We drink (water), and when we drink, the children also drink.”

“It (the glass) has to be colorful, to be a favorite cup, to have a (cartoon) hero on it, to tell (the child): come and drink (water).”

The participating parents reported different strategies on how to decrease the intake of soft drinks and sugared milk drinks in preschoolers. The most commonly mentioned strategies included not buying those beverages, diluting soft drinks with water, using cocoa powder instead of chocolate milk, using fresh fruit juices instead of packed fruit juices or fruit drinks and not drinking soft drinks themselves. However, a few parents in different countries mentioned that it is not necessary to decrease the intake of soft drinks and sugared milk drinks in this age group.

“I don’t have anything against it that, at that age now, he sometimes drinks a glass of lemonade with the meal.”

#### Barriers to changing children’s beverage consumption at home

Parents mentioned some barriers to increase water consumption in preschoolers. For example, children tend to forget that they need to drink when they are playing, children have to be reminded to drink more, the parents cannot control the access to water that the children have at preschool and being consistent and not having any other drinks at home. Belgian low SES parents mentioned that they would have a lot of difficulties with increasing the water intake of their children.

#### Motivational factors to make changes and overcome barriers in the preschool class

Because of the high prevalence of overweight in children, teachers are stimulated to promote healthy drinks in preschool-aged children. Other reported motivational factors included preschoolers’ (sometimes unlimited) consumption of soft drinks at home and teachers being convinced that healthy drinks are better for the children. Barriers for encouraging preschoolers to drink water included parents who sometimes think that teachers are meddling in their family situation, and not all parents want to introduce the preschool rules at home.

“Because if a child tells you he already had coke in the morning… You don’t like to hear that and that’s why it’s important they can drink a healthy drink in school.”

Teachers reported to be motivated to carry out new information into the preschool classroom, when they would receive ready-to-use material. This way, teachers do not lose a lot of time and they can immediately introduce new materials. Furthermore, teachers do not like to use material with a lot of theoretical background, because they already know the theory.

“For instance to make a game in which you can learn what is healthy, what is less healthy, and why. This could, for example, be made with cards. Those could also be used for further discussions. And maybe the game could be performed physically active.”

“A seminar, a two- hour seminar, I think I would not attend… I do not think (of this) as further education; if it was a hundred-hour seminar I would not attend (either), I could not manage.”

#### Beverage consumption at home in six European countries

The most important differences in the six countries were found in the kind of beverages that the children drink. Belgian, German and Spanish parents reported serving sugared milk drinks and fruit juices to their children. However, some parents mentioned that they only serve soft drinks at special occasions. In Bulgaria, Greece and Poland, children only received fruit juices in addition to water and milk. Sometimes, they also drink unsweetened tea in Poland. In Germany, children more often drink (unsweetened) tea, while in Greece and Poland, preschoolers more often drink fruit juices. In all countries however, water was mentioned most. Furthermore, parents would be motivated to increase their child’s water intake and decrease the intake of SSBs, if they received some extra information on the topic from the preschool.

## Discussion

*Parents and teachers perceive children to be sufficiently active at home. They also report that children’s intake of SSB is limited; however, parents and teachers are not factoring in children’s intake of sugared milk drinks and fruit juices because they provide vitamins and minerals and are considered healthy.* In other qualitative studies in which focus group interviews were conducted, similar perceptions on PA were reported by parents [20,26,47] and teachers [19]. However, quantitative studies showed that the activity pattern of preschoolers is characterized by low levels of total PA [6,7] and MVPA [48] and that preschool children have a high mean intake of sugared drinks [11,12] and a low intake of water [13]. Emphasis should be laid on the discrepancy between the caregivers’ perceptions of the healthiness of children’s PA and *beverage consumption* on the one hand and what has been concluded from more objective methods such as accelerometers and dietary records on the other hand. Parallel results were found by Pate et al. [49] in which discrepancy in the perception of caregivers and the actual behaviour was found. As long as parents and teachers perceive preschoolers as physically active, and do not see the need to consume sugared milk drinks and fruit juices only in moderation, they will not take any action to change their children’s behaviour. Once the awareness on this problem is raised (e.g. by letting the children wear an objective measurement device (e.g. an accelerometer), letting the parents fill in a food diary or by using social marketing strategies to spread information about the topic), future interventions targeting both behaviours could include caregivers to positively adapt these EBRBs, at home as well as at preschool. However, in the case of using objective measurement devices, this can only work when parents receive some sort of report so that they can understand what the activity-values mean. After they have received the report, they can set some goals (with or without help from external persons) regarding their child’s PA level. *Although, it is important to keep in mind that this is just one strategy that can be used, which will be more effective in combination with other strategies and even more with changes in the environment.*

Moreover, the parents’ and teachers’ awareness has to be raised on their shared responsibility concerning preschoolers’ PA and *beverage consumption*. In both the home and preschool environment, preschoolers are influenced *to make* (un)healthy decisions on PA and *beverage consumption*[22,33,34]. In the current study, parents and teachers agree that teachers are one of the main role models for the EBRBs (PA and *beverage consumption* in particular) of preschool children. However, according to the teachers, parents should be educated, informed and involved in the promotion of water and PA in preschoolers. *In the study of Hingle et al. (2010), results showed that indirect methods, like bringing home information, do not have a big effect on behaviour change (with respect to nutrition)*[50]*. For a change in dietary outcome, they suggested direct methods to involve the parents. For example, involving them in activities together with the child*[50]*. On the other hand, bringing home information was mentioned by the parents to motivate them to promote healthy drinks and PA. Similar results were found in a qualitative study with parents and teachers from 10- to 12-year olds*[45]*. Again, this shows a discrepancy between results of previous studies and the opinions of parents.*

Furthermore, it is not always clear in interventions what both teachers and parents have to carry out. In the current study, teachers mentioned *that* they only *wanted* material that is ready-to-use. They reported that they do not want any theoretical explanation, because they already have the theoretical background. With ready-to-use material, they do not lose a lot of time and they can use this material immediately in the classroom. In addition, parents want to be involved in what their children learn at preschool but do not always have enough time to attend meetings in the preschool setting. A possible way to increase children’s PA levels is by involving parents and children together in physical activities [23]. For example, one way to increase preschoolers’ PA is the organisation of walking school buses, in which the children come to school in an active way. The walking school bus [51,52] is an opportunity for children to walk to and from school, meanwhile being supervised by adults (mostly parents) and it is timetabled and structured. Currently, this is only established in primary school children. However, the feasibility in preschool children should be investigated. Furthermore, parent–child activities could be organised at the preschool environment, because in the current study, parents reported to be more motivated when they could do activities together with their children at preschool. In these parent–child activities, the messages on PA and *beverage consumption* could be integrated. For example, the preschool could organise a guided walk with stops along the way with healthy drinks and fun and active activities, in which parents and children could participate together. Other examples of parent–child activities are described by Tucker et al. [23] in which parents come to the classroom to take part in classroom-activities together with the children and in which game nights are organised, where the children have the opportunity to compete against their parents in several games. The message on healthy drinks and water consumption can be easily integrated into the parent–child activities. On the other hand, parents reported that they do not always have the time to participate in this kind of activities and teachers reported that it is hard to gather parents at preschool. Therefore, other means of parental involvement could be used. For example, the use of newsletters, tip cards, and bringing home handiworks, could motivate the parents to integrate the healthy messages into the home environment.

The discrepancy between caregivers’ opinions on PA and *beverage consumption* and the actual behaviour, was also noticed across the different countries. German, Greek, Polish, Belgian and Spanish parents agreed that preschoolers are already active enough. Additionally, Polish, Spanish and Belgian teachers reported that preschoolers are very energetic and cannot sit still in preschool. However, in Bulgaria, teachers mentioned that preschool children are not active enough *which might be due to the fact that Bulgarian teachers think that the physical environment/sporting facilities of Bulgarian preschools are not adjusted to the need for PA.* These teachers think it is necessary to increase the PA levels. *Furthermore, parents from the six European countries reported that the intake of SSBs in preschool children is limited. However, objective data on the intake of SSBs in different European countries show the opposite. In Greece, 59.8% of four- to seven-year-old children consumed sugar-added beverages on a daily basis*[53]*. In Belgium, more than 50% of children under four years drink soft drinks or sweetened juices on a daily basis*[54]*. Furthermore, for preschoolers specifically, almost 52% drinks 200 ml or more of sugared drinks every day (including fruit juices)*[55]*. A Spanish study found that when preschoolers drink sugared drinks (soft drinks, extract-based drinks or fruit juices), they drink an amount of 338 ml on a daily basis*[56]*. For the other European countries, no evidence was found on the current levels of sugar-sweetened beverage consumption. However, we can conclude that it is a parental perception that the consumption is low enough. Quantitative numbers show that the intake of sugar-sweetened beverages is high. Furthermore, teachers from the six European countries reported serving different drinks at preschool.* Bulgarian, Polish and Spanish teachers only serve water at preschools, but Belgian and Greek teachers are allowed to serve sugared milk drinks, fruit juices and tea. In Germany, teachers reported to serve diluted fruit juices and unsweetened tea. In summary, it is also important to raise the parents’ and teachers’ awareness in the different countries on the importance of water consumption and enough PA. Furthermore, changing the policies at European preschools could be seen as potential targets for intervention development, since now, there are differences in European countries that can explain differences in preschoolers’ *physical activity and* beverage consumption. *For example, in Belgium and Spain, preschools should include two hours of physical education in the curriculum, which is compulsory in those countries*[57-60]*. This is reflected in the individual centre policies, practices and environments which Belgian and Spanish preschools provide for their children. In those preschools, preschool children receive two hours of physical education each week and they can receive other physical activities on top of the compulsory two hours of physical education. In the other participating European countries, this is not included in the country-level policy, which means that in those countries, preschoolers receive less physical activity compared to preschool children in Belgium and Spain. This can be a possible explanation for the fact that Belgian and Spanish teachers think preschoolers are active enough, while Bulgarian teachers think the preschool children are not active enough.*

### Strengths and limitations

The main strength of this article is that the focus groups were undertaken in six different European countries. Differences and similarities between different European countries were revealed and it was possible to draw multi-cultural and multi-geographical conclusions about parents’ and preschool teachers’ opinions. A second strength of this article is the *undertaking* of focus group interviews with parents and teachers in which their opinions about preschoolers’ PA and *beverage consumption* at home and at preschool were asked. To our best knowledge, no qualitative study has investigated both parents’ and teachers’ *views* on how to change preschoolers’ PA and *beverage consumption*, although the combination is important for the development of interventions, targeting these behaviours simultaneously. Furthermore, executing focus groups with a semi-structured interview guide was also an additional strength of this study.

This study has some limitations. First of all, due to recruitment difficulties, some focus groups were conducted without reaching the conventional group size (e.g. focus group with two teachers instead of a minimum of four teachers), which was required in the focus group protocol. Therefore, the findings might be influenced by the low number of participants in some focus groups. Furthermore, while standardized protocols were used in each European country, different moderators and co-moderators executed the focus group discussions. Consequently, different aspects or themes might have been emphasized in the six different countries. A second limitation was the possibility of selection bias, because participants attended the focus group discussions voluntarily. The most motivated parents and teachers might have participated, whose preschoolers already might have healthy PA and *beverage consumption* behaviours.

## Conclusion

The focus groups revealed an important discrepancy between parents’ and teachers’ perceptions of children’s PA levels and *beverage consumption* compared with what has been found in more objective quantitative studies (e.g. accelerometers, dietary records). As long as parents and teachers do not consider it necessary to increase preschoolers’ PA and to increase their water intake, they will not be convinced to take actions to change these behaviours. Furthermore, their awareness about their shared responsibility has to be raised concerning healthy EBRBs in preschoolers. Both parents and teachers need to carry out different tasks to ensure the development of healthy EBRBs at the home and preschool environment. Parents like to be involved in the things their children learn at preschool, while teachers like to receive ready-to-use material they can easily implement in their classroom.

## Competing interests

The authors have no competing interests to declare.

## Authors’ contributions

MDC conducted the Belgian focus groups, provided feedback on the interview guide, helped with analysing the data, wrote and revised the current manuscript. EDD contributed in conducting the Belgian focus groups and provided suggestions. IDB, BD, CV, EG, YM and GC provided feedback on the interview guide and the standardized protocol before the focus groups were conducted and provided suggestions. KD contributed in conducting and analyzing the German focus groups and provided suggestions. EG contributed in conducting and analyzing the Greek focus groups and provided suggestions. VI provided feedback on the questioning route before the focus groups were conducted and provided suggestions. JMFA contributed in conducting and analyzing the Spanish focus groups and provided suggestions. KZ contributed in conducting and analyzing the Polish focus groups. All authors read and approved the final manuscript.

## Pre-publication history

The pre-publication history for this paper can be accessed here:

http://www.biomedcentral.com/1471-2458/13/278/prepub

## References

[B1] de Onis M Blossner M Borghi E Global prevalence and trends of overweight and obesity among preschool children Am J Clin Nutr 2010 92 5 1257 1264 10.3945/ajcn.2010.29786 20861173

[B2] Hill JO Wyatt HR Reed GW Peters JC Obesity and the environment: where do we go from here? Science 2003 299 5608 853 855 10.1126/science.1079857 12574618

[B3] Kremers SP de Bruijn GJ Visscher TL van Mechelen W de Vries NK Brug J Environmental influences on energy balance-related behaviors: a dual-process view Int J Behav Nutr Phys Act 2006 3 9 10.1186/1479-5868-3-9 16700907 PMC1481572

[B4] van Stralen MM te Velde SJ Singh AS De Bourdeaudhuij I Martens MK van der Sluis M Manios Y Grammatikaki E Chinapaw MJ Maes L EuropeaN Energy balance Research to prevent excessive weight Gain among Youth (ENERGY) project: Design and methodology of the ENERGY cross-sectional survey BMC Publ Health 2011 11 65 10.1186/1471-2458-11-65 PMC3044658 21281466

[B5] Ammerman AS Ward DS Benjamin SE Ball SC Sommers JK Molloy M Dodds JM An intervention to promote healthy weight: Nutrition and Physical Activity Self-Assessment for Child Care (NAP SACC) theory and design Prev Chronic Dis 2007 4 3 A67 17572971 PMC1955393

[B6] Oliver M Schofield GM Kolt GS Physical activity in preschoolers: understanding prevalence and measurement issues Sports Med 2007 37 12 1045 1070 10.2165/00007256-200737120-00004 18027993

[B7] Timmons BW Naylor PJ Pfeiffer KA Physical activity for preschool children–how much and how? Can J Public Health 2007 98 Suppl 2 S122 134 18213943

[B8] Beets MW Bornstein D Dowda M Pate RR Compliance with national guidelines for physical activity in U.S. preschoolers: measurement and interpretation Pediatrics 2011 127 4 658 664 10.1542/peds.2010-2021 21422082 PMC3387888

[B9] Hinkley T Salmon J Okely AD Crawford D Hesketh K Preschoolers’ physical activity, screen time, and compliance with recommendations Med Sci Sports Exerc 2012 44 3 458 465 10.1249/MSS.0b013e318233763b 21900847

[B10] Hawkins SS Law C A review of risk factors for overweight in preschool children: a policy perspective Int J Pediatr Obes 2006 1 4 195 209 10.1080/17477160600943351 17907326

[B11] Huybrechts I Matthys C Vereecken C Maes L Temme EH Van Oyen H De Backer G De Henauw S Food intakes by preschool children in Flanders compared with dietary guidelines Int J Environ Res Public Health 2008 5 4 243 257 10.3390/ijerph5040243 19190355 PMC2672311

[B12] Huybrechts I De Henauw S Energy and nutrient intakes by pre-school children in Flanders-Belgium Br J Nutr 2007 98 3 600 610 10.1017/S000711450773458X 17445352

[B13] Kranz S Siega-Riz AM Herring AH Changes in diet quality of American preschoolers between 1977 and 1998 Am J Public Health 2004 94 9 1525 1530 10.2105/AJPH.94.9.1525 15333309 PMC1448488

[B14] Manios Y Kourlaba G Grammatikaki E Androutsos O Ioannou E Roma-Giannikou E Comparison of two methods for identifying dietary patterns associated with obesity in preschool children: the GENESIS study Eur J Clin Nutr 2010 64 12 1407 1414 10.1038/ejcn.2010.168 20808335

[B15] Manios Y Kourlaba G Kondaki K Grammatikaki E Birbilis M Oikonomou E Roma-Giannikou E Diet quality of preschoolers in Greece based on the Healthy Eating Index: the GENESIS study J Am Diet Assoc 2009 109 4 616 623 10.1016/j.jada.2008.12.011 19328256

[B16] Pate RR Pfeiffer KA Trost SG Ziegler P Dowda M Physical activity among children attending preschools Pediatrics 2004 114 5 1258 1263 10.1542/peds.2003-1088-L 15520105

[B17] Dowda M Pate RR Trost SG Almeida MJ Sirard JR Influences of preschool policies and practices on children’s physical activity J Community Health 2004 29 3 183 196 15141894 10.1023/b:johe.0000022025.77294.af

[B18] Cardon G Labarque V Smits D De Bourdeaudhuij I Promoting physical activity at the pre-school playground: the effects of providing markings and play equipment Prev Med 2009 48 4 335 340 10.1016/j.ypmed.2009.02.013 19236894

[B19] Dwyer GM Higgs J Hardy LL Baur LA What do parents and preschool staff tell us about young children’s physical activity: a qualitative study Int J Behav Nutr Phys Act 2008 5 66 10.1186/1479-5868-5-66 19077255 PMC2634757

[B20] Dwyer J Needham L Simpson JR Heeney ES Parents report intrapersonal, interpersonal, and environmental barriers to supporting healthy eating and physical activity among their preschoolers Appl Physiol Nutr Metab 2008 33 2 338 346 10.1139/H07-195 18347689

[B21] O'Connor JP Temple VA Constraints and facilitators for physical activity in family day care Austr J Early Child 2005 30 4 1 9

[B22] Pocock M Trivedi D Wills W Bunn F Magnusson J Parental perceptions regarding healthy behaviours for preventing overweight and obesity in young children: a systematic review of qualitative studies Obes Rev 2010 11 5 338 353 10.1111/j.1467-789X.2009.00648.x 19780989

[B23] Tucker P van Zandvoort MM Burke SM Irwin JD The influence of parents and the home environment on preschoolers’ physical activity behaviours: a qualitative investigation of childcare providers’ perspectives BMC Publ Health 2011 11 168 10.1186/1471-2458-11-168 PMC3070650 21414218

[B24] Styles JL Meier A Sutherland LA Campbell MK Parents’ and caregivers’ concerns about obesity in young children: a qualitative study Fam Community Health 2007 30 4 279 295 10.1097/01.FCH.0000290541.02834.e0 17873635

[B25] Fees B Trost S Bopp M Dzewaltowski DA Physical activity programming in family child care homes: providers’ perceptions of practices and barriers J Nutr Educ Behav 2009 41 4 268 273 10.1016/j.jneb.2008.01.013 19508932

[B26] Irwin JD He M Bouck LM Tucker P Pollett GL Preschoolers’ physical activity behaviours: parents’ perspectives Can J Public Health 2005 96 4 299 303 16625802 10.1007/BF03405170 PMC5016097

[B27] Bellows L Anderson J Gould SM Auld G Formative research and strategic development of a physical activity component to a social marketing campaign for obesity prevention in preschoolers J Community Health 2008 33 3 169 178 10.1007/s10900-007-9079-z 18196449

[B28] Copeland KA Sherman SN Kendeigh CA Kalkwarf HJ Saelens BE Societal values and policies may curtail preschool children’s physical activity in child care centers Pediatrics 2012 129 2 265 274 10.1542/peds.2011-2102 22218842 PMC3269117

[B29] Copeland KA Sherman SN Khoury JC Foster KE Saelens BE Kalkwarf HJ Wide variability in physical activity environments and weather-related outdoor play policies in child care centers within a single county of Ohio Arch Pediatr Adolesc Med 2011 165 5 435 442 10.1001/archpediatrics.2010.267 21199969 PMC3086945

[B30] Copeland KA Sherman SN Kendeigh CA Saelens BE Kalkwarf HJ Flip flops, dress clothes, and no coat: clothing barriers to children’s physical activity in child-care centers identified from a qualitative study Int J Behav Nutr Phys Act 2009 6 74 10.1186/1479-5868-6-74 19895677 PMC2780978

[B31] Lloyd-Williams F Bristow K Capewell S Mwatsama M Young children’s food in Liverpool day-care settings: a qualitative study of pre-school nutrition policy and practice Public Health Nutr 2011 14 10 1858 1866 10.1017/S1368980011000619 21557874

[B32] Carnell S Cooke L Cheng R Robbins A Wardle J Parental feeding behaviours and motivations. A qualitative study in mothers of UK pre-schoolers Appetite 2011 57 3 665 673 10.1016/j.appet.2011.08.009 21884741 PMC5578399

[B33] Dowda M Pfeiffer KA Brown WH Mitchell JA Byun W Pate RR Parental and environmental correlates of physical activity of children attending preschool Arch Pediatr Adolesc Med 2011 165 10 939 944 10.1001/archpediatrics.2011.84 21646573

[B34] Reilly JJ Low levels of objectively measured physical activity in preschoolers in child care Med Sci Sports Exerc 2010 42 3 502 507 10.1249/MSS.0b013e3181cea100 20068499

[B35] Enrolment in childcare and pre-schoolshttp://www.oecd.org/els/family/37864698.pdf

[B36] Tucker P Irwin JD Sangster Bouck LM He M Pollett G Preventing paediatric obesity; recommendations from a community-based qualitative investigation Obes Rev 2006 7 3 251 260 10.1111/j.1467-789X.2004.00224.x 16866973 PMC5017874

[B37] Trickett EJ Community-based participatory research as worldview or instrumental strategy: is it lost in translation(al) research? Am J Public Health 2011 101 8 1353 1355 10.2105/AJPH.2011.300124 21680920 PMC3134503

[B38] Manios Y Grammatikaki E Androutsos O Chinapaw MJ Gibson EL Buijs G Iotova V Socha P Annemans L Wildgruber A A systematic approach for the development of a kindergarten-based intervention for the prevention of obesity in preschool age children: the ToyBox-study Obes Rev 2012 13 Suppl 1 3 12 22309061 10.1111/j.1467-789X.2011.00974.x

[B39] Compulsory age of starting school in European countries 2010 http://www.nfer.ac.uk/nfer/index.cfm?9B1C0068-C29E-AD4D-0AEC-8B4F43F54A28

[B40] Krueger RA Moderating focus groups (Focus group kit 4): Thousand Oaks 1998 CA: Sage Publishing

[B41] Krueger RA Analyzing and reporting focus group results (focus group kit 6) 1998 Thousand Oaks, CA: Sage Publishing

[B42] Morgan D Planning focus groups (Focus group kit 2): Thousand Oaks 1998 CA: Sage Publishing

[B43] Haerens L De Bourdeaudhuij I Eiben G Lauria F Bel S Keimer K Kovacs E Lasn H Regber S Shiakou M Formative research to develop the IDEFICS physical activity intervention component: findings from focus groups with children and parents J Phys Act Health 2010 7 2 246 256 28872446 10.1123/jpah.7.2.246

[B44] Haerens L De Bourdeaudhuij I Barba G Eiben G Fernandez J Hebestreit A Kovacs E Lasn H Regber S Shiakou M Developing the IDEFICS community-based intervention program to enhance eating behaviors in 2- to 8-year-old children: findings from focus groups with children and parents Health Educ Res 2009 24 3 381 393 10.1093/her/cyn033 18603656

[B45] Van Lippevelde W Verloigne M De Bourdeaudhuij I Bjelland M Lien N Fernandez-Alvira J Moreno L Kovacs E Brug J Maes L What do parents think about parental participation in school-based interventions on energy balance-related behaviours? A qualitative study in 4 countries BMC Publ Health 2011 8 10.1186/1471-2458-11-881 PMC3252293 22112159

[B46] Elo S Kyngas H The qualitative content analysis process J Adv Nurs 2008 62 1 107 115 10.1111/j.1365-2648.2007.04569.x 18352969

[B47] Hinkley T Salmon J Okely AD Crawford D Hesketh K Influences on preschool children’s physical activity: exploration through focus groups Fam Community Health 2011 34 1 39 50 10.1097/FCH.0b013e31820590d6 21135627

[B48] Bornstein DB Beets MW Byun W McIver K Accelerometer-derived physical activity levels of preschoolers: a meta-analysis J Sci Med Sport 2011 14 6 504 511 10.1016/j.jsams.2011.05.007 21684809

[B49] Pate RR McIver K Dowda M Brown WH Addy C Directly observed physical activity levels in preschool children J Sch Health 2008 78 8 438 444 10.1111/j.1746-1561.2008.00327.x 18651931

[B50] Hingle MD O’Connor TM Dave JM Baranowski T Parental involvement in interventions to improve child dietary intake: a systematic review Prev Med 2010 51 2 103 111 10.1016/j.ypmed.2010.04.014 20462509 PMC2906688

[B51] Collins D Kearns RA Walking school buses in the Auckland region: A longitudinal assessment Transport Policy 2010 17 1 1 8 10.1016/j.tranpol.2009.06.003

[B52] Kingham S Ussher S An assessment of the benefits of the walking school bus in Christchurch, New Zealand Transport Res a-Pol 2007 41 6 502 510

[B53] Linardakis M Sarri K Pateraki MS Sbokos M Kafatos A Sugar-added beverages consumption among kindergarten children of Crete: effects on nutritional status and risk of obesity BMC Publ Health 2008 8 279 10.1186/1471-2458-8-279 PMC2525654 18684334

[B54] Eet- en beweeggewoonten bij kinderen en jongerenhttp://www.vigez.be/rubrieken/zoek_op_onderwerp/eet_en_beweeggewoonten_bij_kinderen_en_jongeren.html

[B55] Gezonde voeding voor kleutershttp://www.123aantafel.be/01/123aantafel_01.pdf

[B56] Española de Pediatría CdNdlA Consumo de zumos de frutas y de bebidas refrescantes por niños y adolescentes en España. Implicaciones para la salud de su mal uso y abuso [Consumption of fruit juices and beverages by Spanish children and teenagers: health implications of their poor use and abuse] An Pediatr (Barc) 2003 58 6 584 593 12781116

[B57] Van Cauwenberghe E De Craemer M De Decker E De Bourdeaudhuij I Cardon G The impact of a teacher-led structured physical activity session on preschoolers’ sedentary and physical activity levels J Sci Med Sport 2012 10.1016/j.jsams.2012.11.883 23246443

[B58] Cardon G Van Cauwenberghe E Labarque V Haerens L De Bourdeaudhuij I The contribution of preschool playground factors in explaining children’s physical activity during recess Int J Behav Nutr Phys Act 2008 5 11 10.1186/1479-5868-5-11 18302741 PMC2266778

[B59] De Martelaer K Cools W Samaey C Andries C Van Looy L, Coninx M, Lochtman K De school als bron van mogelijkheden om fysieke actief te zijn in de kleuterfase [The school as a source of opportunities to be physically active in the preschool years] Onderwijsonderzoek: redelijk eigenzinnig?! 2007 Brussels, Belgium: VUBPRESS 191 206

[B60] Pühse U Gerber M International comparison of physical education: Concepts - Problems - Prospects 2005 Oxford: Meyer & Meyer Sport (UK) Ltd 588 603

